# Lactic Fermentation as an Efficient Tool to Enhance the Antioxidant Activity of Tropical Fruit Juices and Teas

**DOI:** 10.3390/microorganisms5020023

**Published:** 2017-05-10

**Authors:** Amandine Fessard, Ashish Kapoor, Jessica Patche, Sophie Assemat, Mathilde Hoarau, Emmanuel Bourdon, Theeshan Bahorun, Fabienne Remize

**Affiliations:** 1 UMR C-95 QualiSud, Université de La Réunion, CIRAD, Université Montpellier, Montpellier SupAgro, Université d’Avignon et des Pays de Vaucluse, ESIROI, 2 rue Wetzell, 97490 Sainte Clotilde, France; amandine.fessard@univ-reunion.fr (A.F.); 113ft0556@nitrkl.ac.in (A.K.); sophie.assemat@cirad.fr (S.A.); mathilde.hoarau@cirad.fr (M.H.); 2 Université de La Réunion, INSERM, UMR 1188, Diabète athérothrombose Thérapies Réunion Océan Indien (DéTROI), plateforme CYROI, 97490 SaintDenis de La Réunion, France; jessica.patche@univ-reunion.fr (J.P.); emmanuel.bourdon@univ-reunion.fr (E.B.); 3 ANDI Centre of Excellence for Biomedical and Biomaterials Research, MSIRI Building, University of Mauritius, 80837 Réduit, Mauritius; tbahorun@uom.ac.mu

**Keywords:** lactic acid fermentation, fruits, pineapple, tea, antioxidant

## Abstract

Tropical fruits like pineapple, papaya, mango, and beverages such as green or black teas, represent an underestimated source of antioxidants that could exert health-promoting properties. Most food processing technologies applied to fruit beverages or teas result in an impairment of inherent nutritional properties. Conversely, we hypothesise that lactic acid fermentation may constitute a promising route to maintain and even improve the nutritional qualities of processed fruits. Using specific growth media, lactic acid bacteria were selected from the fruit phyllosphere diversity and fruit juice, with the latter undergoing acidification kinetics analyses and characterised for exopolysaccharide production. Strains able to ferment tropical fruit juices or teas into pleasant beverages, within a short time, were of particular interest. Strains *Weissella cibaria* 64 and *Leuconostoc mesenteroides* 12b, able to increase antioxidant activity, were specifically studied as potential starters for lactic fermented pineapple juice.

## 1. Introduction

One of the objectives of food research is to investigate new food products that promote health. During the last decade, consumers have increasingly believed that food contributes directly and effectively to health promotion. In this context it has been widely suggested that high intake of fruits and vegetables, nutrient-rich plants and antioxidant-rich beverages like tea and coffee may reduce the risk of degenerative and oxidative-stress related diseases [1,2]. Fresh fruit and vegetables are, however, rapidly perishable; thus the interest for processing which guarantees their safety for consumption. Nevertheless, food processing can trigger undesirable chemical and physical changes [3]. For instance, it has been reported that vitamins and natural antioxidants are rapidly degraded during thermal processing techniques like blanching, cooking, pasteurisation or sterilisation [4], leading to a decrease in antioxidant activity. Significant loss in the vitamin C and flavonoid contents was observed after blanching, air drying, water immersion or boiling of green leafy vegetables, celery and onion bulbs [5,6]. The food industry has thus increasingly considered new alternatives in order to preserve the nutritional and antioxidant properties of foods during food processing.

Lactic acid fermentation is one of the oldest ways of food processing and preservation, liable to keep or increase the safety, nutritional, sensory and shelf-life properties of foods. Lactic acid bacteria (LAB) are a group of gram-positive bacteria, producing lactic acid as the main product of carbohydrate fermentation. During fermentation, LAB produce substances such as bacteriocins, exo-polysaccharides (EPS), aroma compounds, enzymes, B vitamins (mainly folate, riboflavin, cobalamin) or low-calorie polyols (mannitol, sorbitol), which enhance the safety, nutritional quality, sensory properties or antioxidant activities of food [7]. In this vein, *Kimchi*, a traditional fermented mix of vegetables and *Kombucha*, a fermented tea beverage, were associated with antimutagenic, antioxidative, antiaging, cholesterol-lowering activities and prevention against cancer and cardiovascular diseases [8,9]. Several studies have also investigated the ability of lactic acid fermentation to improve the antioxidant properties of fruits and vegetables [10,11,12,13]. An improved preservation of ascorbic acid, glutathione, phenolic compounds and antioxidant activity was observed following lactic acid fermentation of smoothies and tomato juice with *Lactobacillus plantarum*, *Pediococcus pentosaceus* or *Weissella cibaria* [10,14]. Lactic acid fermentation started with *L. plantarum*, *Leuconostoc mesenteroides*, *P. pentosaceus, Lb. delbrueckii* subsp. *lactis*, *Bifidobacterium breve* and *B. thermophilum* increases Vitamin C concentration, phenolic content and antioxidant activity of carrots, French beans, marrows, black beans, pomegranate juice, soy milk and cowpeas [11,15,16,17,18]. Enhancement of antioxidant propensity during fermentation is mainly ascribed to the release of bioactive compounds by LAB [12]. It was reported that LAB were able to increase the antioxidant activity of tea extracts as a result of the modification of tea phenolics [19]. Lactic acid fermentation is thus a promising alternative to expand the range of utilisation of processed foods, while concurrently maintaining and/or boosting nutritional benefits.

In this study, we aimed to develop an attractive fermented beverage through the action of autochthonous lactic acid bacteria isolated from fruit and vegetables grown in Réunion Island. Tropical fruits like pineapple, papaya and mango, together with antioxidant-rich beverages like green and black teas, were chosen as substrates for lactic acid fermentation. The selected raw materials characterised by varying phytochemical composition, more particularly phenolic compounds, were subjected to lactic acid fermentation with the view to identifying pairs of lactic acid bacteria strains and raw material that result in fermented beverages which are technologically feasible, characterised by a pleasant sensory and endowed with increased antioxidant activity.

## 2. Materials and Methods

### 2.1. Bacterial Strain and Culture Media

A total of 28 lactic acid bacteria, isolated from tomatoes (*Lycopersicon esculantum*), papaya (*Carica papaya*) and sliced cabbage (*Brassica oleacera var. capitata*) grown on Réunion Island, were studied to produce a fermented beverage [20].Isolates were stored at −80 °C. These LAB strains were identified by *16S-rRNA*, *pheS* and *recA* gene sequencing. *Lc. citreum* DSM20188, *W. confusa* DSM20196, *W. cibaria* DSM14295, *Lc. pseudomesenteroides* DSM20193 and DSM5625, *Lb. plantarum* DSM2601 were used as reference strains. LAB were grown on MRS (Man Rogosa Sharpe) agar at 30 °C for 72 h. For strain reactivation, one or two colonies or a loop of −80 °C stock were suspended in 9 mL of MRS broth and incubated at 30 °C for 48 h. Thereafter, cultures were homogenised by vortex and 0.2 mL of this suspension was used to inoculate 9 mL of MRS broth. Cell population was estimated by measuring absorbance at 660 nm wavelength.

DNA extraction was performed by using Instagen protocol (Instagen Matrix, Bio-Rad, Marnes-la-Coqiette, France). *Leuconostoc* and *Weissella* spp. were identified by *16S-rRNA* and *pheS* gene sequencing while *Lactobacillus* spp. was identified by *recA* gene sequencing. *16S rRNA* gene sequencing was performed using FD1m and RD1m primers as previously described [20]. The amplification of *pheS* gene was performed according to Naser et al., (2005) and Rubio et al., (2014) [21,22] with some modifications. Primers pheS-21-F (5’-CAYCCNGCHCGYGAYATGC-3’) and pheS-23-R (5’-GGRTGRACCATVCCNGCHCC-3’) were used. The reaction mixture contained buffer 1×, 0.2 mmol·L^−1^ dNTP, 0.5 µmol·L^−1^ each primer, 1.5 mmol·L^−1^ MgCl_2_ and 0.04 U·µL^−1^ *Taq* polymerase (Thermo Fisher Scientific, Villebon, France). The PCR reaction was performed in a 45 µL volume with 10 µL of DNA solution. The thermal program consisted of 5 min at 95 °C; 35 cycles of 1 min at 95 °C, 30 s at 56 °C, 1 min 15 s at 72 °C; 7 min at 72 °C. In order to differentiate *Lb. plantarum/paraplantarum/pentosus*, *recA* gene sequencing was performed according to Torriani et al., (2001) [23]. Multiplex PCR was performed using primers PlanF (5’-CCGTTTATGCGGAACACCTA-3’), ParaF (5’-GTCACAGGCATTACGAAAAC-3’), PentF (5’-CAGTGGCGCGGTTGATATC) and PREV (5’-TCGGGATTACCAAACATAAC-3’). The reaction mixture contained buffer 1×, 1.5 mmol·L^−1^ MgCl_2_, 0.25 µmol·L^−1^ ParaF, PentF and PREV, 0.12 µmol·L^−1^ PlanF, 12 µmol·L^−1^ dNTP and 0.025 U·µL^−1^ *Taq* polymerase. The thermal program consisted of 3 min at 94 °C; 30 cycles of 30 s at 94 °C, 10 s at 56 °C, 30 s at 72 °C; 5 min at 72 °C. The products of positive PCRs (a 318 bp amplicon for *Lb. plantarum* spp. or a 107 pb amplicon for *Lb. paraplantarum* spp.) were purified with a GelElute Extraction kit (5Prime, Duscher SA, Brumath, France).

PCR products were sequenced by the Sanger method with FD1m, pheS-21F, PlanF and ParaF primers as previously described [20].

### 2.2. EPS Production

EPS production was determined using MRS sucrose (40 g·L^−1^) agar as previously described [20].

### 2.3. Kinetics of Acidification

Kinetics of acidification was performed as previously described using MRS broth as culture media [20]. Each culture was performed in triplicate. Parameters of acidification lag time, pH_min_, V_M_, t_M_, pH_M_ were determined from dpH/dt = f(t) curves as previously described. Lag time (λ) was the time corresponding to an initial pH variation of less than 0.1. Minimal pH (pH_min_) was the final constant pH value. Maximum acidification rate (V_M_), time to reach the maximum acidification rate without the lag phase (t_M_), and pH at maximal acidification rate (pH_M_) were determined from dpH/dt = f(t) curves.

### 2.4. Growth in Apple Juice

Growth in apple juice was performed in sterile 96-well microplates. LAB were grown for 48 h at 30 °C in 9 mL MRS broth. Bacterial suspension was centrifuged at 8000× *g* for 5 min. The supernatant was removed and the cell pellet was washed twice with sterile distilled water and re-suspended in 9 mL of water. Then, 20 µL of this bacterial suspension were inoculated in 180 µL of clarified apple juice (Carrefour, Sainte Clotilde, France). This juice contained 19.4 g·L^−1^ glucose, 63.1 g·L^−1^ fructose and 9.7 g·L^−1^ sucrose. The control medium corresponds to apple juice plus 20 µL of distilled water. Microplates were incubated at 30 °C for 96 h. OD at 660 nm was measured every 2 h (Infinite M200 Pro, Tecan, Lyon, France). Results were expressed as log (OD_96_/OD_0_). Apple juice culture was performed in triplicate for each strain.

### 2.5. Preparation of Substrate

Commercial pineapple juice was used directly for all experiments. Pineapple juice contained 41.1 g·L^−1^ glucose, 41.8 g·L^−1^ fructose and 42.1 g·L^−1^ sucrose. Papayas or mangoes were washed and peeled. The seeds or stones were removed and mixed in order to obtain a purée. The latter was pasteurised for 5 min at 75 °C using a water bath with temperature monitoring and cooled to room temperature before fermentation. For the tea infusion, sugar cane syrup was added to distilled water in order to reach 10% sucrose concentration. One tea bag was infused for 5 min in 250 mL of boiling sweetened water and the infusion was cooled at room temperature before fermentation.

### 2.6. Fermentation

LAB strains were reactivated for 48 h at 30 °C in 9 mL of MRS broth. Then, 48 h reactivated cultures were used to inoculate 50 mL pre-culture of MRS broth with an initial optical density at 660 nm of 0.05. Pre-cultures were incubated at 30 °C for 48 h. After centrifugation at 8000× *g* for 5 min, the cell pellet was washed twice with sterile distilled water and re-suspended in 20 mL of sterile water in order to have a concentrated cell culture. Optical density at 660 nm was measured and volume of inoculation was calculated in order to have an initial OD of 0.05. With the appropriate volume, 40 mL of pineapple juice or green tea or black tea or 40 g of papaya or mango purée were inoculated with the concentrated culture. Substrates were incubated at 30 °C without agitation. Thereafter, fermented products were incubated at −20 °C for 15 min to stop the fermentation. Fermentation was performed in triplicate for each strain.

### 2.7. Qualitative Sensory Analysis and Determination of Fermentation Optimal Time

In order to determine optimal time for fermentation and select a reduced number of potential starters, pineapple juice was subjected to fermentation at 30 °C for two days or four days with each of the 35 strains and placed at 4 °C for 24 h before sensory analysis. A qualitative sensory analysis was carried out by five non-trained people. Substrates were scored from “excellent” to “unsatisfactory” for colour, aroma, appearance and uniformity. Strains were scored as (++) if global sensory analysis was excellent, (+) for a good global sensory analysis and (−) for an unsatisfactory global sensory analysis.

### 2.8. Shelf-Life Determination

After 48 h of fermentation, control juice and started pineapple juice with *W. cibaria* 64 and *Lc. pseudomesenteroides* 12b were placed at 4 °C for 16 days. After inoculation, following fermentation or periodically over storage, 1 mL of non-started and started juices were suspended into 9 mL sterile peptone water solution and homogenised. Lactic acid bacteria population was determined on MRS agar at 30 °C for 48–72 h. Yeast counts were determined on glucose chloramphenicol agar at 25 °C for 5 days. Total mesophilic bacteria counts were determined on nutritive agar at 30 °C for 72 h. Enterobacteria population was calculated from colonies observation on VRBG agar after 48 h at 37 °C. The pH, sugar concentration and antioxidant activity were also monitored during the same period.

### 2.9. Sugar Content Determination

The Miller method was used to determine reducing sugar concentration [24], with glucose as standard. Sugar concentration (sucrose, glucose and fructose) were also analysed by HPLC (UltiMate 3000 system 6-1, Dionex, Thermo Fisher Scientific, Villebon, France) with a refractometry detector. For the HPLC column, milliQ water was used as the mobile phase and injection volume was 20 µL. The analysis was performed isocratically at 0.6 mL·min^−1^ flow rate at 80 °C with a Ca USP L19 (250 × 4.0 mm) column (Hi-Plex, Agilent Technologies, Les Ulis, France). Calibration curves were prepared from sucrose, glucose and fructose standards and were used for the quantification. The concentration range for each standard was 0.5 g·L^−1^ to 0.03125 g·L^−1^. The correlation coefficient *r*² for the calibration curves were 0.9987, 0.9993 and 0.9860 for sucrose, glucose and fructose, respectively. Pineapple and apple juice were 100-fold diluted for HPLC analysis.

### 2.10. Sensory Analysis

Sensory profile was carried out by a panel of nine trained members. After fermentation, non-started pineapple juice and fermented juice started with the strains *W. cibaria* 64 or *Lc. pseudomesenteroides* 12b were refrigerated, encoded with three-digit random numbers and served (20 mL). Products were served anonymously with randomisation, and the analysis was performed in triplicate. Control and fermented juices were scored from 0 to 10 for colour, odour, texture, flavour, taste and general quality. The sensory attributes used for this study were: yellow, green, and orange for the colour; pineapple, soft, acid, fermented, fruity, yoghurt and sweet for the odour; smoothness, fluidity, watery and syrupy for texture; acid, sweet, bitter and salt for the flavour; fresh, pineapple, fruity, yoghurt, spicy, astringent, hot, fermented, sweet-sour, and sparkling for the taste.

The fermented juice started with strains *W. cibaria* 64 or *Lc. pseudomesenteroides* 12b were also evaluated by 37 untrained panellists (17–50 years old). The panel was composed of 59.5% women and 40.5% men. Most of the panellists were under 20 years old or 20–30 years old. Most of the panellists are used to drinking pineapple juice at least once per month or not at all (51% and 32%, respectively). The two fermented pineapple juices were placed at 4 °C for two days before hedonic analysis. The samples were identified with random three-digit codes, distributed in portions of 20 mL into transparent cups and served in a randomised order. The panellists were asked to evaluate the overall acceptability, colour, odour, taste, pineapple taste and texture. Scales from 1 (dislike extremely) to 10 (like extremely) were used for overall acceptability, odour and taste. Scales from −2 (very repulsive, too discreet, or too fluid) to 2 (very attractive, too intense or too viscous) were used to evaluate the colour, pineapple taste and texture, respectively.

### 2.11. Determination of Total Phenolic Content (TPC)

Determination of TPC was performed using the Folin–Ciocalteu method with gallic acid as standard as previously described [20]. Pineapple juice and tea were centrifuged at 8000 g for 5 min at 4 °C. The supernatants were collected and stored at −20 °C until use. For papaya and mango purée, phenolic compounds were extracted according to Septembre-Malaterre et al., (2016) [25] with some modifications. Purée (6 g) was mixed to 30 mL of distilled water and incubated at 4 °C for 90 min. Then after, samples were centrifuged at 4000× *g* for 20 min at 4 °C. Results are expressed in grams of gallic acid equivalent (GAE) per litre for pineapple juice, green and black tea. For mango and papaya purée, results were expressed in milligrams of GAE per 100 g of puree.

### 2.12. 1,1-Diphenyl-2-Picrylhydrazyl (DPPH) Free-Radical Scavenging Activity (RSA)

DPPH radical scavenging activity was performed as previously described [20] with some modifications. Gallic acid and water were used as positive and negative control, respectively. All samples were 10-fold diluted for this assay. Results were expressed in % DPPH according to the formula:

$$\%\;\mathrm{of}\;\mathrm{DPPH}\;\mathrm{reduced}\;=\;\frac{\left(OD\;Control-OD\;sample\right)}{OD\;Control}\;\times 100$$

### 2.13. Red Blood Cell Haemolysis Assay

Red blood cell haemolysis assay was performed as previously described [20]. Blood samples were obtained from healthy volunteers. Pineapple juices and papaya pulps were 100-fold and 50-fold diluted, respectively. Cell lysis was followed by the decrease of absorbance at 450 nm at 10-min intervals for 16 h at 37 °C (Infinite M200 Pro, Tecan, Lyon, France). Results are expressed as half-time of haemolysis (HT-50), determined from GraphPad Prism 5.01 with a Boltzman sigmoidal nonlinear regression as previously described.

### 2.14. Low Density Lipoprotein (LDL) Oxidation

Blood samples were obtained from normolipidemic healthy volunteers. Red blood cells and plasma were separated by centrifugation at 2000× *g* for 5 min. For each preparation, at least 30 mL of pooled plasma was used as starting material. Plasma density was adjusted to 1.22 g·mL^−1^ by adding solid KBr. Three millilitres of KBr-loaded plasma was layered in the bottom of a centrifuge tube (Beckman centrifuge, Beckman Coulter, Villepinte, France). The loaded plasma fraction was gently overlaid with five millilitres of a KBr solution (density 1.063 g·mL^−1^). Finally, the KBr solution was overlaid with one millilitre of milliQ water. LDLs isolation was performed by ultracentrifugation (24 h, 29,000 rpm, 4 °C) in a Beckman Coulter centrifuge equipped with a SW 41 Ti rotor. This step resulted in five layers from top to bottom: Residual water solution; Low-density lipoprotein fraction (density lower than 1.063 g·mL^−1^); Residual KBr solution; High-density lipoprotein fraction; Residual plasma solution. LDL was gently collected and dialyzed against PBS 1× (pH 7.4). LDLs were assayed for protein concentration by the Bradford method using Bovine Serum Albumin (BSA) as the standard. LDLs were sterile filtered and stored at 4 °C in the dark for no longer than 15 days. LDL oxidation was determined by measuring conjugated diene formation at 234 nm, as previously described [20] with some modifications. Final LDL protein concentration in each well was 100 µg·mL^−1^. Pineapple juices and papaya pulps were 500-fold and 50-fold diluted, respectively. Results are expressed in half-time oxidation (V-50) in min, obtained from GraphPad Prism 5.01 with a Blotzman sigmoidal nonlinear regression, as previously described.

### 2.15. Statistics

The software XLSTAT (Addinsoft, Paris, France) was used for all statistical analyses. Significant effects of factors were detected with a Fisher test (*p*-value < 0.0001). Significant differences versus a control or by pairs were tested with Dunnett’s and Ryan, Einot, Gabriel, Welch q (REGWQ) tests respectively.

## 3. Results and Discussion

### 3.1. EPS Formation and Acidification Kinetics

In this study, a set of strains originating from tropically grown plant phyllosphere and belonging to several species were used. The strains were classified under the genera *Lactobacillus*, *Leuconostoc*, *Weissella*, *Lactococcus* and *Fructobacillus*. Among those, 13 species were represented. Strains were characterised by determination of EPS production ability and acidification kinetic parameters.

EPS formation was observed for all *Leuconostoc* spp., *W. confusa* and *W. cibaria* strains, with liquid or creamy colony phenotypes depending on the strain (Table 1). These three groups were previously reported as EPS producers [26]. Other *Weissella* species and other genera did not exhibit EPS production under these conditions.

Acidification kinetic parameters were obtained from the curves of pH determination over time in MRS (Table 2). Latency time varied greatly, from 2.5 h for *W. confusa* 17 to 26.1 h for *Lc. pseudomesenteroides* 89. Except for those extreme behaviours, half the latency times were in the range 9–14.7 h. Another parameter that varied greatly was the maximal acidification rate, V_M_. Extreme values were 84 mUpH·h^−1^ for *W. paramesenteroides* 37 and 377 mUpH·h^−1^ for *W. confusa* DSM20196. Half of the strains exhibited V_M_ in the range 155 to 220 mUpH·h^−1^. Lastly, T_M_, which is the time required for V_M_ after latency, exhibited a variation coefficient of 13%. This time was comprised between 2.8 h and 8.8 h, respectively, for *W. confusa* DSM20196 and *Lc. pseudomesenteroides* 12b.

The minimal pH range was narrow, with extreme values of 3.6 and 4.5. The lowest minimal pH was observed for two *Lb. plantarum* strains, 17a and DSM2601, followed by *Lb. plantarum* 75, *Lc. mesenteroides* 5 and *Fb. tropaeoli* 77. The highest final acidification pH was observed for *Lc. pseudomesenteroides* 79, followed by *Lc. citreum* 2, *W. confusa* 59 and *W. soli* 58. Dispersion of minimal pH was low with a variation coefficient of 4%. A very low dispersion was also observed for values of pH_M_, which is the pH corresponding to the maximal rate of acidification. More than half of strains exhibited a pH_M_ between 5.0 and 5.1.

A remarkable diversity of behaviour was observed within the group of strains. Acidification kinetic parameters were not related to species or strain origin. This diversity is an opportunity regarding possible application of these strains as starters as it could lead to diverse fermentation profiles. Moreover, a starter preparation requires us to define conditions and duration to obtain strains with a good fitness.

### 3.2. Growth in Apple Juice

**Figure 1:**
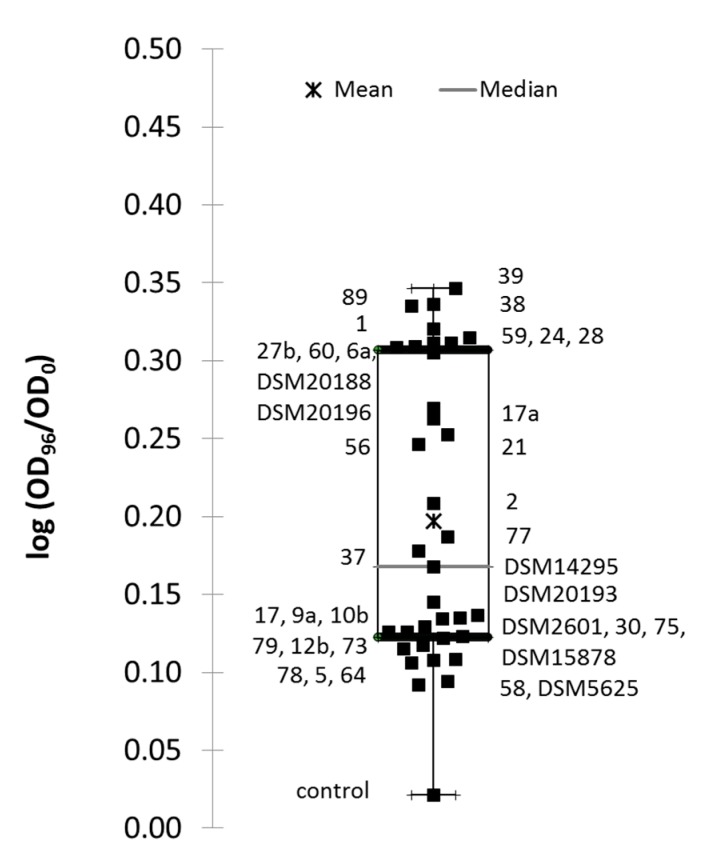
Boxplot of means of growth yield expressed as log(OD_96h_/OD_0h_) for strains cultivated into apple juice. Each black square corresponds to the mean growth yield for the indicated strain. Lowest and upper bars correspond to extreme values of yield. The empty box corresponds to second and third quartiles, star to the general mean and line in the box to the median value.

Apple juice was chosen as it was shown to be a substrate which supported growth of *Lactobacillus* spp. strains [27]. Moreover, this substrate resulted in large differences of growth performances within the genus. The growth yield of the 34 strains was determined from turbidity change in apple juice over the 96-hour culture. The growth yield was significantly different from the non-inoculated control, for 19 strains, as assayed by Dunnett’s unilateral test (*p*-value being comprised between 0.05 and <0.0001). Pairwise comparison showed that three strains, *Lc. pseudomesenteroides* 89, *Lc. mesenteroides* 1 and *Lc. mesenteroides* 28 exhibited the highest growth yield. Other strains with significant growth ranked from the highest to the lowest growth yield were *Lc. pseudomesenteroides* 39, *Lc. lactis* 24, *Lc. mesenteroides* 6a, *Lc. pseudomesenteroides* 27b, *W. confusa* 38, *W. confusa* 59, *Lc. pseudomesenteroides* 60, *Lb. plantarum* 17a, *W. cibaria* 21, *Lc. pseudomesenteroides* 56, *Lc. citreum* 2, *Fb. tropaeoli* 77 and *W. paramesenteroides* 37, in addition to three reference strains, *Lc. citreum* DSM20188, *W. confusa* DSM10196 and *W. cibaria* DSM14295 (Figure 1). All the *W. confusa* and the *Lc. mesenteroides* strains were within the 19-strain group, whereas no *Lactobacillus* spp. strain was present in this group. The other 16 strains exhibited a much lower growth yield than the 19-strain group. They were grouped in a narrow range of growth yield close to the first quartile value (Figure 1).

### 3.3. Starter Selection Based on Hedonic Evaluation

In order to make a selection of starters, each strain was used to start pineapple juice fermentation and hedonic evaluations of fermented juice were performed after two days and four days of fermentation (Table 3). From the results obtained, strains 2, 73, 75, 77, 78, 79, 89, 9a, DSM2601, DSM5625 and DSM20188, were not taken on for further assays. From retained strains, fermentation assays were conducted over two days at 30 °C, then subsequently cooled down before storage. Potential starters were screened for their capacity to increase antioxidant activity of substrate over-fermentation. Fermented products that exhibited off flavours were not included in the antioxidant assay determination.

### 3.4. Lactic Fermentation of Black or Green Tea Infusion

**Figure 2:**
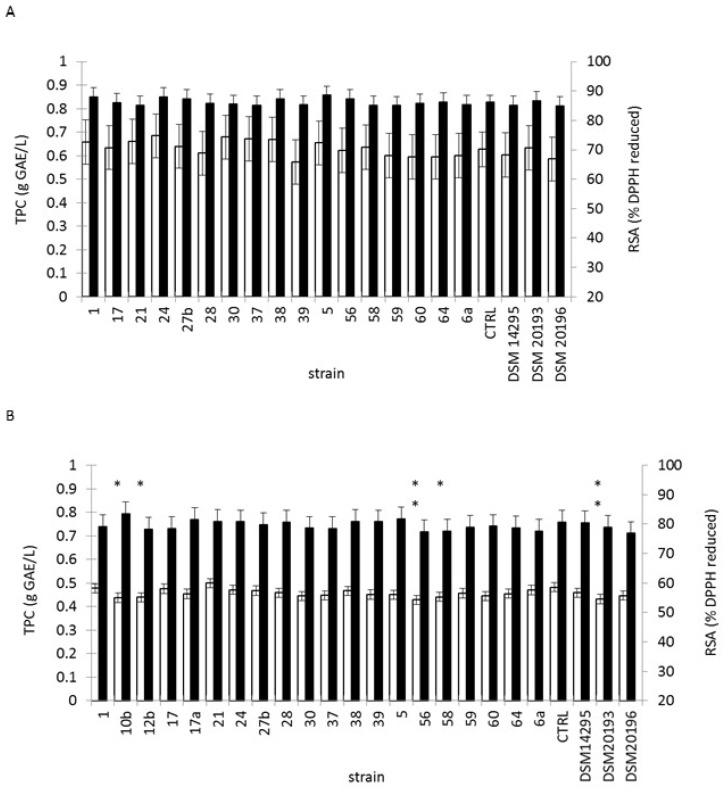
Total phenolic content and radical scavenging activity in tea (**a**) green tea and (**b**) black tea. White bar: Total polyphenol content (TPC) of material fermented for 48 h with the indicated starter strain. Black bar: Radical scavenging activity (RSA) of material fermented for 48 h with the indicated starter strain. Control, labelled CTRL, corresponds to incubated juice. ** *p* < 0.01, * *p* < 0.05 from Dunnett’s test versus control.

Control green and black tea infusions differed by their TPC and antioxidant activity (Figure 2A,B). TPC were 0.63 ± 0.03 and 0.48 ± 0.04 g GAE·L^−1^ and RSA were 85.9% ± 1.7% and 80.7% ± 1.7%, respectively, for green and for black tea. This is consistent with tea characteristics, as green tea has been reported to have a higher antioxidant potency than black tea [28,29,30,31]. TPC may vary by time of infusion, teabag and type of tea [32]. Green and black teas differ in their composition but also in their manufacturing process. After harvest, leaves for green tea are rapidly processed in order to inactivate enzymes and avoid polyphenol oxidation. On the contrary, black tea leaves are first withered in order to concentrate polyphenols. The withered leaves are fermented and in that process the polyphenols are oxidised [33]. As a consequence, green tea is richer in catechins, the most important ones being (−) epigallocatechin gallate, (−) epicatechin gallate, (+) catechin, (−) epicatechin and (−) epigallocatechin, whereas black tea contains high levels of theaflavins and thearubigins, the resulting oxidised and polymerised forms of catechins [19,34]. For green tea infusions, neither TPC nor RSA was modified by the fermentation process (Figure 2A). A mean total polyphenol content of 0.63 g GAE·L^−1^ was observed, with a variation coefficient of 20%. RSA ranged between 75.6 and 90.7% whatever the nature of sample, fermented or not. For black tea infusions, RSA was not affected by fermentation with any of the strains and ranged between 63.3 and 90.4% (Figure 2B). Concerning TPC, a significant effect was observed for some fermented product. However, the change corresponded to a decrease of the content. The average value of total polyphenols was 0.45 g GAE·L^−1^ (Figure 2B). *Lactobacillus* spp. have been shown to increase the antioxidant activity of tea extracts by catabolism of tea phenolics [19]. However, to the best of our knowledge, no study has reported on the lactic acid fermentation of tea infusions by *Weissella* or *Leuconostoc* spp.

### 3.5. Fermentation of Tropical Fruit Preparations

Mango pulp, papaya pulp and pineapple juice were used as substrates for fermentation, started with previously selected starters.

**Figure 3:**
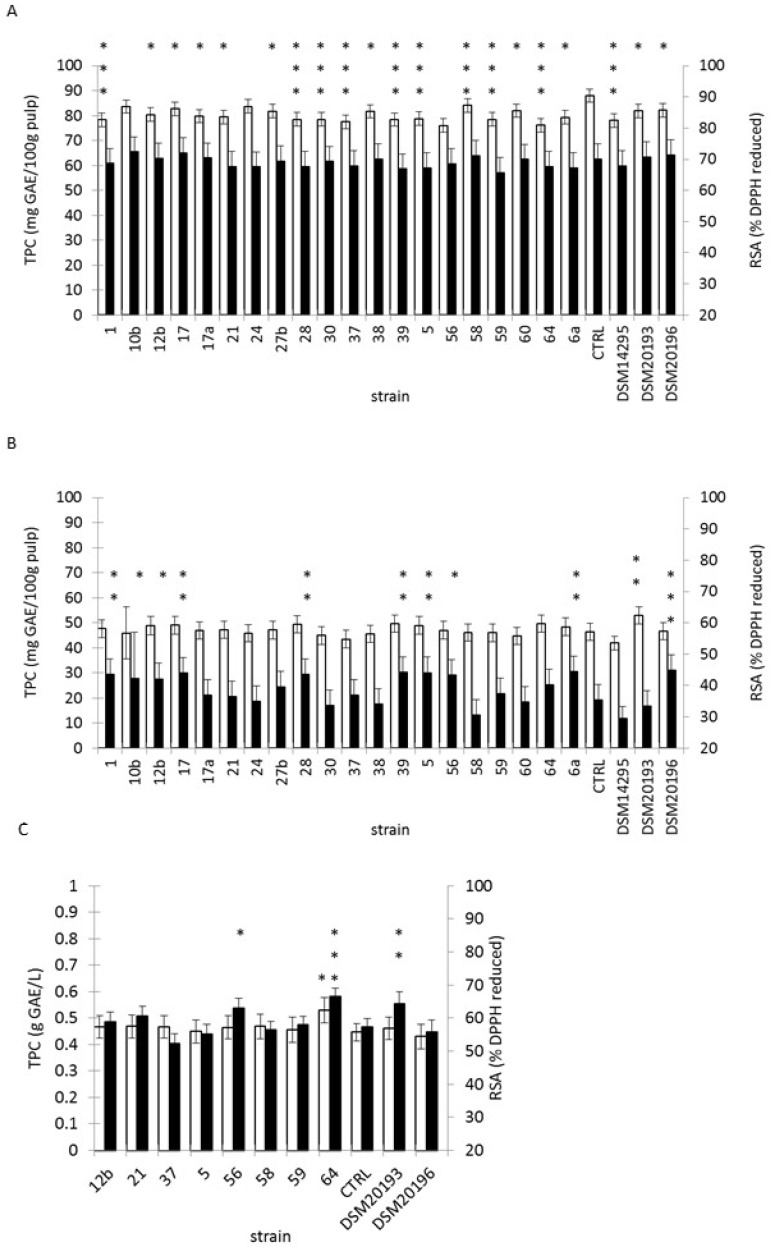
Total phenolic content and radical scavenging activity of fruits: (**A**) mango pulp; (**B**) papaya pulp; (**C**) pineapple juice. White bar: Total polyphenol content (TPC) of material fermented for 48 h with the indicated starter strain. Black bar: Radical scavenging activity (RSA) of material fermented for 48 h with the indicated starter strain. Control, labelled CTRL, corresponds to incubated juice. *** *p* < 0.0001, ** *p* < 0.01, * *p* < 0.05 from Dunnett’s test versus control.

Mango pulp fermentation resulted in the absence of changes in RSA (Figure 3A). This activity was comprised between 45.3 and 80.3%, with a mean at 69.1%. On the opposite, TPC was decreased during fermentation of several mango pulp samples. Nevertheless, the phenolic content determined for mango pulp was higher than those reported in the literature (41.1 mg GAE/100g and 78.3 mg GAE/100g, by Chen et al., 2014 [35] and by Septembre-Malaterre et al., 2016 [25], respectively). Mango fruit was shown to be rich in hydroxybenzoic acids (gallic acid, vanillic acid, protocatechic acid), in chlorogenic acid and in glucoside hydroxybenzoic derivatives (gallic acid-O-hexoside, syringic acid-O-hexoside) [25,36].

Fermentation of papaya pulp resulted for several starter strains in changes in TPC and in RSA, compared to the control conditions (Figure 3B). Dunnett’s test pointed out a significant increase of TPC, when fermentation was started with DSM20193 (*p*-value 0.027). The same test applied to RSA, showed increases for fermented papaya started with DSM10196, 6a, 39, 5, 17, 28, 1, 56, 10b and 12b with *p*-values raking from 0.001 to 0.05, respectively. In fermented papaya pulp, TPC ranged between 32.8 and 64.8 mg GAE/100g of pulp. RSA was in the extreme range of 2.9%–57.9% with a mean at 38.6%. These values were notably below the ones observed for tea. The phenolic level detected for papaya was close to that reported by Septembre-Malaterre et al., (2016) [25]: 33.4 and 41.3 mg GAE/100g for papaya Colombo and for papaya Solo respectively.

Non-fermented pineapple juice exhibited a TPC of 0.45 ± 0.02 g GAE·L^−1^ and a RSA of 57.3% ± 1.3%, which is consistent with previous data [25]. For pineapple juice, TPC exhibited a significant increase as detected by the Dunnett’s unilateral test, only for juice started with *W. cibaria* 64 (*p*-value 0.045) (Figure 3C). Fermented juice started with strain 17 exhibited a marked decrease in TPC and antioxidant activity (data not shown). A significant increase of antioxidant activity as measured with the DPPH assay was also observed for pineapple juice started with strain 64 (*p*-value < 0.0001). With this assay, other significant increases of activity were detected for juice started with *Lc. pseudomesenteroides* DSM10193 (*p*-value 0.005) and *Lc. pseudomesenteroides* 56 (*p*-value 0.013).

Papaya pulp and pineapple juice share several common features. They are rich in hydroxycinnamic acids (ferulic acid, caffeic acid, *p*-coumaric acid and sinapic acid) and in glucoside hydroxycinnamic derivatives [25,37,38]. By contrast, these molecules are not the main polyphenolics in tea and in mango pulp. The observed increase of phenolic content during lactic acid fermentation may result from the depolymerisation or hydrolysis of phenolic compounds [12]. Several enzymes, like β-glucosidase, tannase, feruloyl-esterase or phenolic acid decarboxylase have been characterised in *Lactobacillus* spp [18,39,40]. Tannase and β-glucosidase were detected in *W. paramesenteroides*, *Lc. mesenteroides* and *Lc. fallax* while feruloyl esterase was observed in *Leuconostoc* spp. [41,42]. Formation of exopolysaccharides, glutathione, superoxide dismutase and catalase was also involved in the increase of antioxidant activities during lactic acid fermentation [12]. EPS production by *W. confusa*, *W. cibaria* and *Leuconostoc* spp. may partly explain the observed changes but RSA modulation was not species-dependent.

A subset of pair’s food material—strain was assayed for red blood cell protection from free-radical induced haemolysis and for LDL protection from copper-induced oxidation activity. Papaya pulp and pineapple juice were used as they were the only substrates in which increases of TPC or RSA were observed during fermentation. Moreover, these two substrates exhibited the lowest RSA values, which might facilitate the detection of RSA increase. Fermentation was started with strain 64, DSM20193, 56 or 12b for both substrates and with strains 17, 28, 1, 56 or 10b for papaya pulp only. Haemolysis half-time values increased from 269 ± 20 min for control cells to 302 ± 24 min for 50-fold-diluted papaya pulp and to 282 ± 24 for 100-fold-diluted pineapple juice (Table 4). The haemolysis half-time of pulp started with strain 1 was significantly higher than for control cell (*p*-value 0.032). Besides this observation, the half-time was reduced for pulp started with 10b versus incubated pulp (*p*-value 0.028) and for juice started with 12b versus incubated juice. For LDL oxidation assay, half-time with control cells was significantly lower than with 500-fold-diluted incubated pineapple juice and juice started with 56, 12b or 64 (Table 4). LDL oxidation half-time data was higher for 500-fold-diluted pineapple juice than for 50-fold-diluted papaya pulp. No significant difference between fermented and unfermented pulp was observed. The effects observed with DPPH assay were not reflected by haemolysis inhibition neither by LDL oxidation inhibition. This discrepancy can be explained by the differences in methods that do not trigger the same types of molecular mechanisms and/or involve different radicals or pro-oxidants and/or are conditional on the experimental time [43]. A further analysis of polyphenols, but also EPS and peptide composition changes during fermentation, would be necessary to understand these observations.

### 3.6. Changes in Microbial Counts, pH, Sugar and RSA Over-Fermentation and Refrigerated Storage

The pH during fermentation showed an increase from 3.4 to 4.0 for 64-started juice and to 3.5 for 12b-started juice. The pH became constant after 16 days of storage. This pH increase could result from decarboxylation of malic or citric acids, the main organic acids in pineapple, which have two and three carboxyl groups, respectively [44,45]. Reducing the sugar level decreased over-fermentation, starting from 79 g·L^−1^ and reaching 60.5 and 61.8 g·L^−1^, respectively, for juice started with 64 and with 12b. HPLC sugar analyses showed different patterns. For juice started with 64, glucose was the main sugar consumed with concentration decreasing from 39.4 g·L^−1^ to 28.4 g·L^−1^ over-fermentation. Sucrose content decreased from 36.6 g·L^−1^ to 32.5 g·L^−1^ and fructose content decreased from 44.7 g·L^−1^ to 40.2 g·L^−1^. The resulting product is 20% poorer in sugars compared to the non-fermented juice, which is another interesting nutritional property. For juice started with 12b, only glucose was consumed, resulting in a content of 33.3 g·L^−1^. Surprisingly, after 16 days of storage at 4 °C, the reducing sugar level was 79.2 and 71.4 g·L^−1^, respectively, for juice started with 64 and with 12b. The concentration of glucose, fructose and sucrose was 38.1, 46.4 and 48.4 g·L^−1^, respectively, for juice started with 64. For juice started with 12b, the concentration of glucose, fructose and sucrose was 36.6, 40.7 and 40.9 g·L^−1^, respectively. Altogether, an increase of reducing sugar content and of glucose, fructose and sucrose was observed for 64-started juice over shelf-life. This observation stems probably from the hydrolysis of polysaccharides or ester bounds releasing sugars or, in a lesser extent, from the synthesis of aldehydes or ketones which contain reducing extremities. The synthesis of different aldehydes and ketones was previously observed for *W. cibaria* and numerous *Leuconostoc* species in sourdough and cheeses [46,47]. This observation further warrants the requirement for analysis of composition changes during fermentation and storage. During storage, the RSA of started juice increased by 34% and by 29%, with strains 64 and 12b, respectively.

LAB count at inoculation was 1.0×10^5^ cfu·mL^−1^. It increased to 7.5×10^5^ cfu·mL^−1^ over-fermentation with strain 64 and decreased during refrigerated storage to 1.5×10^5^ cfu·mL^−1^. For 12b, a marked increase was evident during fermentation and the counts reached 7.5×10^7^ cfu·mL^−1^. An additional increase up to 1.6×10^8^ cfu.mL·mL^−1^ was observed during storage. Neither enterobacteria nor yeasts and moulds were detected in started juices and total bacterial counts corresponded exactly to LAB counts.

On the whole, 16 days of refrigerated storage did not significantly alter the microbial quality of fermented juices and preserved antioxidant properties.

### 3.7. Sensory Profile and Fermented Juice Quality Evaluation

**Figure 4:**
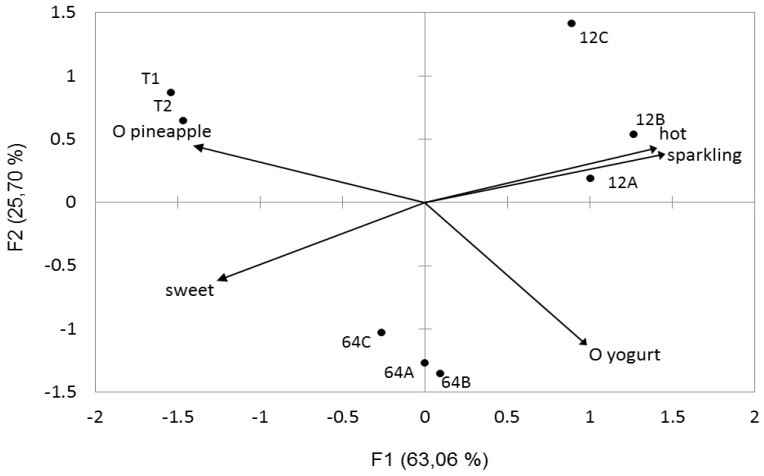
Principal component analysis of sensory profiles of pineapple juice non-fermented or started with 64 and 12b strains. T1 and T2, pineapple juice; 64A, 64B, 64C, juice started with strain 64; 12A, 12B, 12C, juice started with strain 12b. Arrows correspond to eigenvectors for indicated descriptors.

Pineapple juice started with *W. cibaria* 64 or with *Lc. pseudomesenteroides* 12b was chosen for sensory analysis. Pineapple juice was characterised by a high acidity and a sweet flavour, but also by fluidity, smoothness and freshness. Yogurt odour and taste were significantly more marked in started juice compared to control juice (strain 64 *p* < 0.001; strain 12b *p* < 0.05). Other sensory attributes did not significantly differ between the control and the 64-started juice. Hot and sparkling tastes were more present in pineapple juice started with strain 12b compared to the control and to 64-started juice (*p* < 0.001). Moreover, 12b-started juice was characterised by a more pronounced sweet and sour taste compared to the control juice (*p* < 0.05). Lastly, the pineapple character was significantly lower in 12b started juice compared to control juice (*p* < 0.01). These characteristics result in a significantly reduced overall quality for 12b started juice compared to non-fermented juice (*p* < 0.05). Interestingly, PCA analysis showed a very clear grouping of assays according to a limited number of sensory attributes (Figure 4). Yogurt character and sweet taste reflect *W. cibaria* 64-started juice properties, whereas *Lc. pseudomesenteroides* 12b-started juices were characterised by hot and sparkling tastes and non-fermented juice by its marked pineapple taste.

Altogether, started juice was liked with global quality scores of 5.5 and 5.1, respectively, for juices started with 64 and 12b. The non-started juice was more appreciated, with a score of overall quality of 6.9.

**Figure 5:**
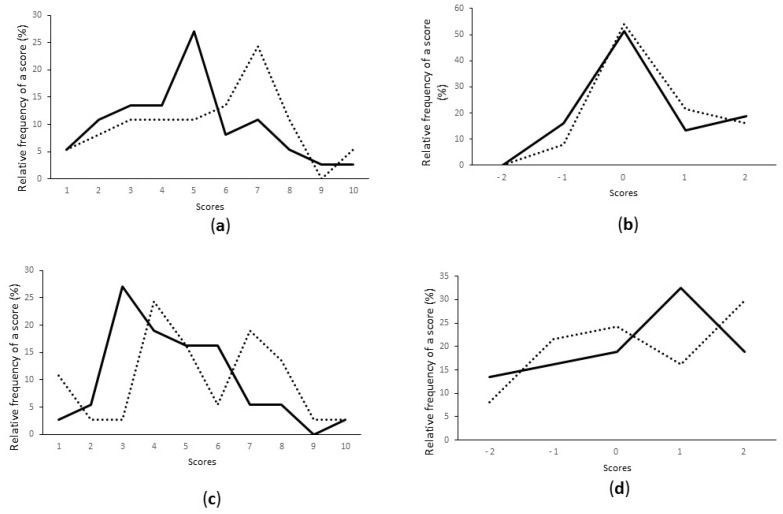

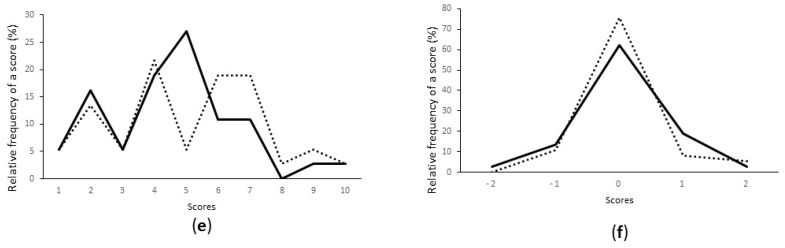
Hedonic sensory analysis: (**a**) overall acceptability; (**b**) colour; (**c**) odour; (**d**) pineapple taste; (**e**) taste; (**f**) texture. Light dotted line: juice started with 12b; black line: juice started with 64.

Hedonic test scores showed that fermented pineapple juice started with strain 12b was more appreciated than that started with strain 64, but the difference was not significant (Figure 5). Colour, taste, pineapple flavour and texture evaluation did not show differences according to the starter strain. The only difference was for odour, slightly more appreciated in 12b-started juices.

## 4. Conclusions

A set of 34 strains from 13 species with highly diverse characteristics was screened for the ability to start lactic fermentation of juice, pulps and tea infusions and to lead to pleasant beverages with preserved antioxidant properties. Two strains were particularly investigated, 64 and 12b. The fermented juices were globally appreciated and exhibited peculiar sensory characteristics.

*W. cibaria* 64 produced EPS, had moderate acidification ability and grew poorly in apple or pineapple juice. Pineapple juice fermented with this strain exhibited a significant increase in TPC and RSA, which was maintained during refrigerated storage. However, other antioxidant activity determination assays did not succeed in detecting changes. *Lc. pseudomesenteroides* 12b also produced EPS but exhibited a slow acidification profile. Its growth in apple juice was poor but good in pineapple juice. With this strain as starter, an increase in antioxidant activity was noted in fermented papaya pulp but remained unchanged in fermented pineapple juice. In a green tea infusion or mango pulp, fermentation with this strain resulted in decreased TPC. These differences probably reflect different enzymatic activities that modulate the composition of fruit juice over-fermentation. The investigation of metabolite changes in fruit juice during lactic fermentation with these strains and the determination of enzymatic activities involved in polyphenolic compounds’ hydrolysis would lead to a better understanding of the observed properties of fermented juices. Lastly, fruit lactic fermentation with strains from those species is an unusual event, though these species are often detected in fermented foods of plant origin [48,49,50] and would thus require safety studies. In particular, this might entail that biogenic amine formation and infectious potential would have to be carefully investigated [51,52].

## Figures and Tables

**Table 1:** Identification, origin and exo-polysaccharide (EPS) phenotype of LAB strains used in the study.

Strain	Species	Origin	Production EPS	Aspect
1	*Lc. mesenteroides*	Papaya	+	Liquid
2	*Lc. citreum*	Sliced cabbage	+	Creamy
5	*Lc. mesenteroides*	Sliced cabbage	+	Liquid
17	*W. confusa*	Sliced cabbage	ND *	ND *
21	*W. cibaria*	Sliced cabbage	+	Creamy
24	*Lc. lactis*	Sliced cabbage	+	Creamy
28	*Lc. mesenteroides*	Sliced cabbage	+	Creamy
30	*W. cibaria*	Sliced cabbage	+	Liquid
37	*W. paramesenteroides*	Papaya	-	-
38	*W. confusa*	Papaya	+	Creamy
39	*Lc. pseudomesenteroides*	Papaya	+	Liquid
56	*Lc. pseudomesenteroides*	Sliced cabbage	+	Creamy
58	*W. soli*	Sliced cabbage	−	-
59	*W. confusa*	Sliced cabbage	+	Liquid
60	*Lc. pseudomesenteroides*	Sliced cabbage	+	Creamy
64	*W. cibaria*	Sliced cabbage	+	Creamy
73	*Lb. paraplantarum*	Sliced cabbage	−	-
75	*Lb. plantarum*	Sliced cabbage	−	-
77	*Fb. tropaeoli*	Papaya	−	-
78	*Lc. pseudomesenteroides*	Papaya	+	Liquid
79	*Lc. pseudomesenteroides*	Papaya	+	Liquid
89	*Lc. pseudomesenteroides*	Sliced cabbage	+	Creamy
10b	*W. cibaria*	Tomato	+	Creamy
12b	*Lc. pseudomesenteroides*	Tomato	+	Liquid
17a	*Lb. plantarum*	Tomato	−	-
27b	*Lc. pseudomesenteroides*	Papaya	+	Liquid
6a	*Lc. mesenteroides*	Papaya	+	Creamy
9a	*Lc. citreum*	Tomato	+	Creamy
DSM14295	*W. cibaria*	Kimchi	+	Creamy
DSM20188	*Lc. citreum*	ND *	+	Creamy
DSM20193	*Lc. pseudomesenteroides*	Sugar cane juice	+	Liquid
DSM20196	*W. confusa*	Cane sugar	+	Creamy
DSM2601	*Lb. plantarum*	Pickled cabbage	−	-
DSM5625	*Lc. pseudomesenteroides*	Commercial starter	+	Creamy

* ND, not determined.

**Table 2:** Parameters of acidification (mean ± SD).

Strain	Species	Latency (h)	pH min	Vm (mUpH·h^−1^)	Tm (h)	pHm
1	*Lc. mesenteroides*	25.3 ± 7.3	3.9 ± 0.4	205 ± 91	4.5 ± 0.2	5 ± 0.1
2	*Lc. citreum*	12.0 ± 0.8	4.3 ± 0.2	154 ± 2	4.1 ± 0.5	5.1 ± 0.1
5	*Lc. mesenteroides*	12.3 ± 5.1	3.7 ± 0.2	218 ± 47	5.4 ± 1.2	4.9 ± 0.2
17	*W. confusa*	2.5 ± 0.0	4.1 ± 0	211 ± 33	3.6 ± 0.3	5.1 ± 0.2
21	*W. cibaria*	9.0 ± 3.6	4 ± 0.1	180 ± 38	4.1 ± 0.9	5 ± 0.1
24	*Lc. lactis*	6.6 ± 3.3	4.1 ± 0.1	170 ± 36	3.3 ± 0.7	5.3 ± 0.2
28	*Lc. mesenteroides*	17.3 ± 5.3	3.9 ± 0.2	212 ± 68	4.7 ± 0.5	5 ± 0.2
30	*W. cibaria*	8.4 ± 0.2	4.1 ± 0.1	180 ± 72	4.3 ± 0.1	5 ± 0.2
37	*W. paramesenteroides*	12.0 ± 3.7	3.9 ± 0.1	84 ± 9	4.8 ± 0.3	5 ± 0
38	*W. confusa*	16.2 ± 2.0	4.1 ± 0.1	144 ± 25	4.3 ± 0.9	5 ± 0.1
39	*Lc. pseudomesenteroides*	7.5 ± 3.1	4 ± 0.2	196 ± 63	4.9 ± 1.1	5 ± 0.1
56	*Lc. pseudomesenteroides*	12.2 ± 4.6	4 ± 0.2	258 ± 44	5 ± 0	5 ± 0
58	*W. soli*	18.2 ± 4.1	4.3 ± 0.2	128 ± 20	8.6 ± 0.4	5 ± 0.5
59	*W. confusa*	16.1 ± 2.6	4.3 ± 0.1	126 ± 51	4.8 ± 0.5	5.2 ± 0.1
60	*Lc. pseudomesenteroides*	14.1 ± 0.6	4 ± 0.1	257 ± 55	3.2 ± 0.6	5 ± 0
64	*W. cibaria*	10.8 ± 2.0	4 ± 0.2	192 ± 11	3.3 ± 0.2	4.9 ± 0.2
73	*Lb. paraplantarum*	11.2 ± 1.4	3.8 ± 0.1	234 ± 5	5 ± 0.4	5 ± 0
75	*Lb. plantarum*	8.8 ± 3.4	3.7 ± 0.1	225 ± 83	4.2 ± 0.5	5.1 ± 0.1
77	*Fb. tropaeoli*	13.3 ± 6.9	3.7 ± 0.1	263 ± 28	4.9 ± 0.3	5 ± 0
78	*Lc. pseudomesenteroides*	14.7 ± 0.6	4 ± 0.2	206 ± 48	4.3 ± 0.4	4.9 ± 0.1
79	*Lc. pseudomesenteroides*	17.8 ± 7.1	4.5 ± 0.4	133 ± 12	4.6 ± 0.6	5.1 ± 0.2
89	*Lc. pseudomesenteroides*	26.1 ± 7.1	3.8 ± 0.3	165 ± 23	6.1 ± 1.5	4.8 ± 0.1
10b	*W. cibaria*	9 ± ND *	4.1 ± ND	100 ± ND	6.3 ± ND	5 ± ND
12b	*Lc. pseudomesenteroides*	8.6 ± 2.7	4 ± 0.3	138 ± 19	8.8 ± 2.8	5 ± 0.2
17a	*Lb. plantarum*	7.3 ± 4.7	3.6 ± 0.2	237 ± 35	4.6 ± 1.1	5 ± 0.3
27b	*Lc. pseudomesenteroides*	10 ± 3.9	3.9 ± 0.2	149 ± 13	8.1 ± 1.5	4.9 ± 0.3
6a	*Lc. mesenteroides*	10.6 ± 5.2	4 ± 0	184 ± 15	4.1 ± 0.8	5.1 ± 0.2
9a	*Lc. citreum*	16.5 ± 0.7	3.8 ± 0.1	243 ± 40	4.1 ± 0.2	5 ± 0
DSM14295	*W. cibaria*	10.5 ± 1.4	4 ± 0.1	200 ± 6	3.7 ± 0.6	5.1 ± 0.1
DSM20188	*Lc. citreum*	10 ± 1.4	4 ± 0.1	184 ± 45	8.1 ± 1.6	5.2 ± 0.1
DSM20193	*Lc. pseudomesenteroides*	13.1 ± 3	4.1 ± 0.6	221 ± 73	4.1 ± 0.5	5.1 ± 0.1
DSM20196	*W. confusa*	14.7 ± 3	4 ± 0.1	377 ± 31	2.8 ± 0.5	5 ± 0.1
DSM2601	*Lb. plantarum*	7.2 ± 1.6	3.6 ± 0	193 ± 52	5.6 ± 0.6	4.8 ± 0
DSM5625	*Lc. pseudomesenteroides*	10.5 ± 1.9	3.9 ± 0.1	159 ± 36	5.9 ± 0	4.9 ± 0.1

* ND, not determined.

**Table 3:** Sensory attributes and optimal time fermentation. (−) not acceptable, (+) acceptable, (++) pleasant odour.

Sensory Properties of Pineapple Juice After Fermentation			
Strain	Species	2 Days	4 Days
5	*Lc. mesenteroides*	+	+
17	*W. confusa*	++	++
21	*W. cibaria*	+	+
37	*W. paramesenteroides*	++	++
56	*Lc. pseudomesenteroides*	+	+
58	*W. soli*	+	++
59	*W. confusa*	++	++
12b	*Lc. pseudomesenteroides*	+	+
1	*Lc. mesenteroides*	+	-
24	*Lc. lactis*	++	-
28	*Lc. mesenteroides*	+	-
30	*W. cibaria*	−	++
38	*W. confusa*	−	++
39	*Lc. pseudomesenteroides*	−	+
60	*Lc. pseudomesenteroides*	−	+
10b	*W. cibaria*	+	-
17a	*Lb. plantarum*	−	+
27b	*Lc. pseudomesenteroides*	−	++
6a	*Lc. mesenteroides*	++	-
64	*W. cibaria*	+	+
DSM14295	*W. cibaria*	−	+
DSM20193	*Lc. pseudomesenteroides*	−	++
DSM20196	*W. confusa*	+	+
DSM2601	*Lb. plantarum*	−	-
2	*Lc. citreum*	−	-
73	*Lb. paraplantarum*	−	-
75	*Lb. plantarum*	−	-
77	*Fb. tropaeoli*	−	-
78	*Lc. pseudomesenteroides*	−	-
79	*Lc. pseudomesenteroides*	−	-
89	*Lc. pseudomesenteroides*	−	-
9a	*Lc. citreum*	−	-
DSM20188	*Lc. citreum*	−	-

**Table 4:** Haemolysis inhibition and LDL oxidation inhibition by fermented papaya pulp or pineapple juice started with different strains (mean ± SD). * Correspond to a *p*-value < 0.05. Different letters correspond to significant differences.

Condition	Half-Time for Haemolysis (Min)		Half-Time for Ldl Oxidation (Min)	
CONTROL CELLS	269 ± 20		28.1 ± 4.2	B
PAPAYA PULP	302 ± 24		45.1 ± 5.1	AB
PAPAYA/STRAIN 1	340 ± 27	* vs control cells		
PAPAYA/STRAIN 10B	257 ± 94	* vs pulp		
PAPAYA/STRAIN 12B	264 ± 70		52.3 ± 23.0	AB
PAPAYA/STRAIN 28	280 ± 40			
PAPAYA/STRAIN DSM20193	319 ± 20			
PAPAYA/STRAIN 56	259 ± 46		52.0 ± 15.0	AB
PAPAYA/STRAIN 64	279 ± 12		54.2 ± 28.2	AB
PAPAYA/STRAIN 17	278 ± 7			
PINEAPPLE JUICE	282 ± 24		71.7 ± 9.4	A
PINEAPPLE/STRAIN 12B	208 ± 72	* vs juice	75.9 ± 12.7	A
PINEAPPLE/STRAIN DSM20193	273 ± 73			
PINEAPPLE/STRAIN 56	234 ± 79		75.7 ± 12.4	A
PINEAPPLE/STRAIN 64	289 ± 47		54.2 ± 28.2	A
